# In-Person Caretaker Visits Disrupt Ongoing Discomfort Behavior in Hospitalized Equine Orthopedic Surgical Patients

**DOI:** 10.3390/ani10020210

**Published:** 2020-01-27

**Authors:** Catherine Torcivia, Sue McDonnell

**Affiliations:** 1 Department of Clinical Studies, University of Pennsylvania School of Veterinary Medicine, New Bolton Center, 382 W Street Road, Kennett Square, PA 19348, USA; 2 Havemeyer Equine Behavior Lab and Clinic, Department of Clinical Studies, University of Pennsylvania School of Veterinary Medicine, New Bolton Center, 382 W Street Road, Kennett Square, PA 19348, USA; suemcd@upenn.edu

**Keywords:** equine, pain assessment, orthopedic surgery, discomfort behavior

## Abstract

**Simple Summary:**

In 24-h video-recorded samples of 20 hospitalized equine orthopedic surgery patients, ongoing discomfort behavior conspicuously diminished or stopped altogether, when a caretaker approached or interacted with the horse, and then resumed after the caretaker’s departure. For all 20 patients, the degree of reduction was potentially important to clinical management decisions. Current state-of-the-art equine clinical composite pain scoring rubrics rely on observations of discomfort behavior in combination with physiologic measures, such as heart rate, respiratory rate, body temperature, and gut motility. All of these are typically assessed concurrently during a visit by a caretaker. This raises concern that discomfort in equine patients is routinely underestimated in ways that might compromise patient welfare. While this is especially of concern for veterinary hospitals, this natural characteristic of horses to show little indication of discomfort or disability in the presence of predators is also likely to delay recognition of disease in horses in general.

**Abstract:**

Horses have evolved to show little indication of discomfort or disability when in the presence of potential predators, including humans. This natural characteristic complicates the recognition of pain in equine patients. It has been our clinical impression that, whenever a person is present, horses tend to “perk up” and ongoing discomfort behavior (DB) more or less ceases. The objective of this study was to quantitatively evaluate and describe this effect. For each of 20 orthopedic surgical patients, continuous 24-h video was reviewed to record all occurrences of DB during a caretaker visit (3.23 to 7.75 min), for comparison to the hour preceding as well as the hour following when undisturbed. The mean ± S.E. DB observed per minute during the preceding and following hours, respectively, were 1.65 ± 0.17 and 1.49 ± 0.22. The difference was not significant (*p* > 0.05). In contrast, mean DB per minute during the visit was 0.40 ± 0.11. This was significantly lower than during both the preceding and following hours (*p* < 0.0001). All 20 patients expressed fewer observable DB per minute during the visit, with a mean reduction of 77.4% ± 0.17%. For 30% of these patients, ongoing DB ceased altogether during the visit. These findings confirm our clinical impression that caretaker visits interrupt DB, resulting in under-appreciation of discomfort.

## 1. Introduction

Horses have evolved to show little indication of discomfort or disability when in the presence of potential predators, including humans. This natural characteristic complicates the recognition and management of pain in horses. To assist veterinary clinicians in identifying potential sources of discomfort behavior in patients, we routinely evaluated 24-h continuous video samples of stalled horses [1]. It has been our clinical impression that, regardless of a patient’s ongoing discomfort behavior, whenever people approach or interact, the horse “perks up” and discomfort behavior more or less ceases. Although this apparent tendency for interruption of observable discomfort behavior in the presence of people has been mentioned in the literature [1,2], it does not appear to be widely appreciated in equine clinical practice. It is particularly concerning that the current state-of-the-art objective pain scoring protocols that include behavior assessments [3,4] are often done within the context of an in-person caretaker visit, which requires direct interaction with the horse. Therefore, it is important to critically evaluate our clinical impression of interruption of ongoing discomfort behavior in the presence of people. Accordingly, the objective of this study was to quantitatively evaluate the effect of a caretaker visit on ongoing discomfort behavior of equine patients.

## 2. Materials and Methods

### 2.1. Case Selection

This study was conducted using recently recorded and archived 24-h video of client-owned orthopedic surgical patients hospitalized at the University of Pennsylvania New Bolton Center’s large animal hospital. All animal procedures for obtaining video recordings were approved by the University of Pennsylvania Institutional Animal Care and Use Committee, protocol #806321. Records were reviewed to identify 20 cases for which their continuous 24-h VHS video sample included a caretaker visit (either to observe and examine or to administer treatment), which was both preceded and followed by one hour of no disturbance (no one interacting with the horse and no indication from the video of the presence of staff or other disturbance in the barn). These patients included 7 females, 4 intact males, and 7 castrated males of various breeds (10 Thoroughbred, 6 Warmblood, 2 Standardbred, 1 Arabian, 1 Quarter Horse) and ages (range: 1 to 21 years, median: 7.5 years).

Horses were housed in individual stalls, together in hospital barns, each with 10 or more stalls holding other horse patients on both sides of a central aisle. Open grates on stall fronts facing the aisle enabled patients to see and hear other horses, as well as any patient care activities in the barn. Time of day of the samples varied among patients. For 8 patients, their sample occurred during late morning or early afternoon (11:00–18:00). For the remaining 14 patients, their sample occurred during evening or overnight hours (18:00–06:00). Caretakers involved in these visits were trained equine veterinary hospital staff, unfamiliar to any of the patients included in this study.

### 2.2. Data Collection

The visit duration ranged from 3.23 min to 7.75 min. This included the time from the caretaker entering until leaving the stall. From the video view it was not always apparent when the caretaker first approached stall-side, or when they left the barn. To ensure that the horse was truly undisturbed during the preceding and following hours, 5 min of recorded video, each, immediately before and after the caretaker entered and exited the stall were excluded from the evaluation.

The video of the visit as well as the preceding and following hours was reviewed in real time to record on a time base all occurrences of observed discomfort behavior (DB) during those intervals (for list and descriptions of DB, see, Appendix A Table A1). Discrete behavioral events (e.g., shifting weight, difficulty rising, stepping in place, kicking out or back, romping/bucking, flinching, stretching, rotational shaking head or body, head tossing, looking at affected area, swatting, abandoning recumbency or elimination attempt) or brief series (e.g., lifting/holding limb up, pawing, rolling, abbreviated weaving, sympathetic surge resolution signs, yawning bout, autogrooming, focusing ears caudally, swishing tail, circling/pacing, nibbling aimlessly, fidgeting) were each counted as a single DB. Abnormal locomotion or postures (e.g., non-physiologic locomotion, pointing, prolonged resting of limb, standing base narrow or wide, leaning against objects, atypical recumbency, changing activities frequently, conservative movement) were counted as 1 DB for each minute in which it was observed. The video viewing technician (CT) was a veterinarian with advanced training in equine behavior, particularly experienced with pain recognition in horses, both for research and clinical purposes. The technician was highly experienced with our laboratory’s equine discomfort ethogram, which defines 65 specific observable behavioral responses associated with discomfort related to various systems in horses. This equine discomfort ethogram, along with video examples for each DB is in preparation for an expected 2020 publication by the authors.

### 2.3. Data Analysis

For each patient, the number of specific DB responses per minute during the visit, as well as during the hour preceding and the hour following the visit was calculated. Differences in mean DB per minute preceding, during, and following the visit were compared using two-tailed dependent *t*-test procedures or the non-parametric equivalent Wilcoxon Signed Rank Test for non-normally distributed data (Shapiro Wilk Test). The software program Statistix 10 (Analytical Software, Tallahassee, FL, USA) was used for data analysis.

## 3. Results

**Figure 1:**
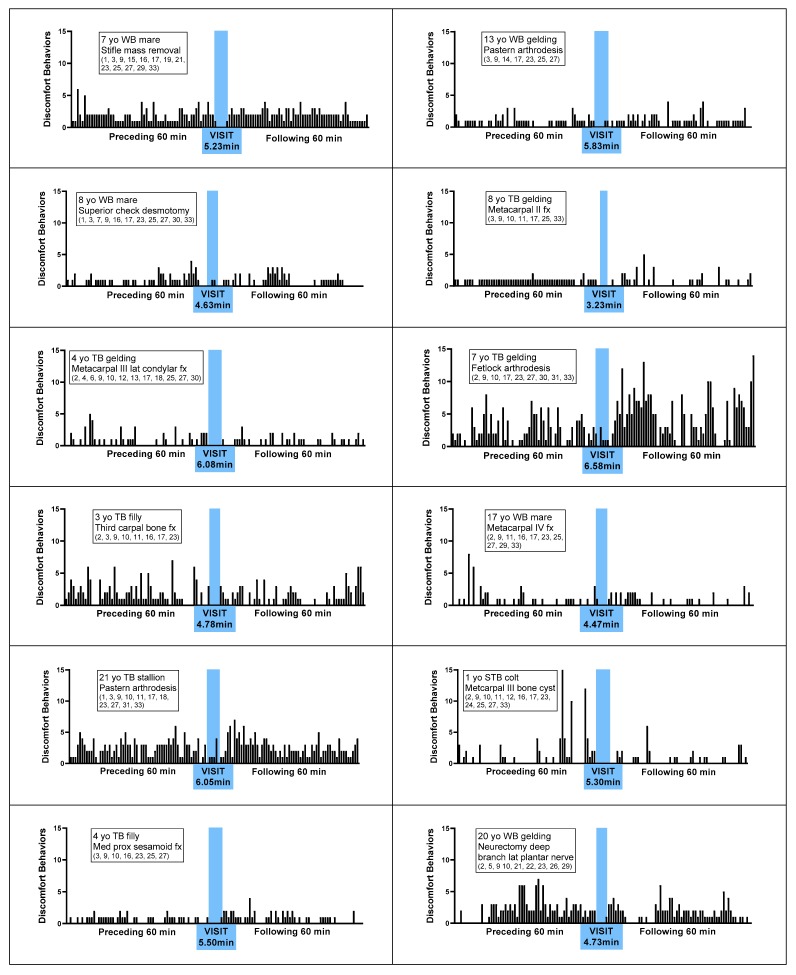

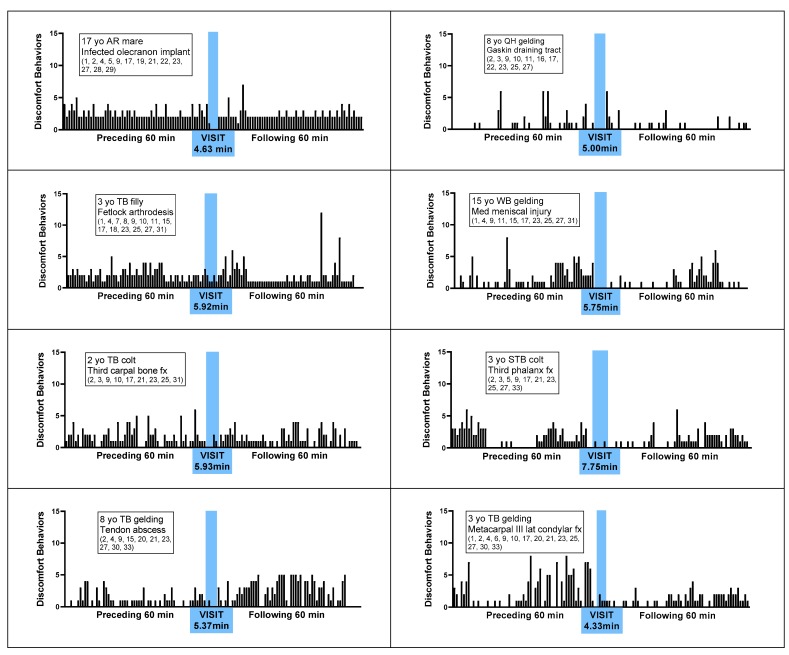
Discomfort behaviors per minute displayed by 20 horses with various orthopedic surgical conditions, during the 60 min preceding a caretaker visit, during a caretaker visit, and 60 min following a caretaker visit. Time of caretaker visit is highlighted in blue. Numbers in parentheses refer to the particular set of discomfort behaviors observed for the horse, as described in Appendix A Table A1. Abbreviations: yo = years old, WB = warmblood, TB = Thoroughbred, STB = Standardbred, QH = Quarter Horse, AR = Arabian, fx = fracture, prox = proximal, med = medial, lat = lateral.

**Figure 2:**
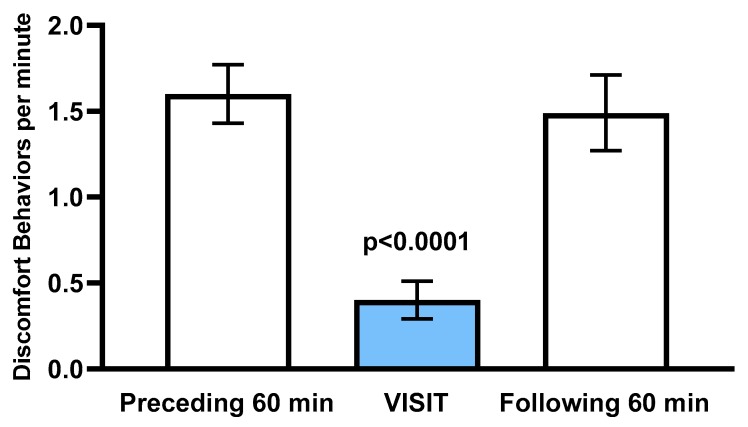
Mean ± S.E. discomfort behaviors per minute during the 60 min preceding a caretaker visit, during a caretaker visit (blue), and 60 min following a caretaker visit, n = 20 patients.

Figure 1 represents the number of DBs observed during each minute of the hour preceding the visit, during the visit, and following the visit, for each of the 20 patients. Observations included a total of 33 distinct behavioral indictors of discomfort, as defined and illustrated in Appendix A Table A1. For all 20 patients, fewer DB per minute were observed during the visit, with a mean ± S.E. reduction of 77.4% ± 0.17 % (range 24% to 100%), compared to the mean DB per minute observed during the preceding and following hours. For 6 (30%) of the 20 patients, ongoing observed DB ceased altogether during the visit. As illustrated in Figure 2, the overall mean ± S.E. number of DB per minute during the preceding and following hours, respectively, were 1.60 ± 0.17 and 1.49 ± 0.22. The difference was not significant (*p* > 0.10). In contrast, mean DB per minute during the visit was 0.40 ± 0.11. This was significantly lower than during both the preceding hour and the following hour (*p* < 0.0001 in each case).

For each patient, the probability of an interval equal to the duration of the visit resulting in a DP per minute equal to or less than that observed during the visit was manually calculated. For 5 of the 20 patients (25%), the probability was less than 0.0001. For another 5 patients (25%), the probability was between 0.03 and 0.05. For the remaining 10, the probability ranged from 0.07 and 0.42. This meant that in the case of at least 10 of these subjects, one would not expect the lower DB during the time of the visit to have occurred by chance alone.

## 4. Discussion

These data clearly confirm our clinical impression that ongoing discomfort behavior of hospitalized equine patients is conspicuously interrupted when people approach or interact. This sample involved only orthopedic surgery patients. Our experience has been that similar disruption of ongoing discomfort behavior occurs with discomfort resulting from sources other than musculoskeletal. While especially of welfare concern with hospitalized patients, this tendency to show little indication of discomfort or disability in the presence of potential predators likely similarly delays recognition of injury or disease in horses in general. Further, we are concerned that this significant reduction in observable discomfort behavior in the presence of a caretaker or visitor, the resulting potential underestimation of discomfort, and the potential impact on clinical case management, are not widely appreciated in equine practice.

Another equine pain study [4] serendipitously observed less discomfort behavior when observers were present, however, did not report attributing it to caretaker presence. These researchers evaluated behavior of hospitalized equine patients, pre- and post-arthroscopy. Their design included 24-h time lapse video to evaluate activity time budgets, as well as periodic stall-side direct observation (24, 12, and 2 h pre-surgery, and 2, 4, 6, 12, 24, and 48 h post-surgery). Each 15 min caretaker visit included two 5-min periods of continuous behavior observation separated by a 5-min interval. The authors commented that certain discomfort behaviors, for example, restlessness and weight shifting, that had been seen on the video evaluation at other times had not actually occurred during the particular times of direct observation, even when the observer was positioned at a distance. They apparently attributed this to chance. They concluded that analysis of activity time budgets over a period of hours was more sensitive than periodic brief direct observation. They indicated that time budget analysis was impractical for non-research situations, and suggested that for clinical situations, more frequent in-person observation might increase the likelihood of observing those behaviors. Based on over 35 years of experience (SM) evaluating video in a hospital situation along with these current data, we hypothesized that increased frequency of in-person visits would not effectively improve recognition of ongoing discomfort behavior. Our observations have been that regardless of visit frequency, discomfort behavior continues to be interrupted whenever people approach.

Although all patients in this sample displayed a lower rate of DB during the caretaker visit than during either the preceding or following hour, the degree of reduction varied considerably (24–100%). Obviously, there are many intrinsic and environmental factors that affect expression of discomfort [2] (for review). Further study involving a larger sample of patients would be necessary to evaluate variation associated with, for example, severity of procedure/condition, individual animal characteristics (age, breed, sex), time of day, caretaker characteristics (familiarity and demeanor), as well as other characteristics of the environment at the time. Of course, an a priori designed sampling schedule and patient selection criteria would be required to systematically address these factors.

Current state-of-the-art composite pain scoring protocols for horses [5,6] include observation of behavior in combination with a set of physiologic measures, such as heart and respiratory rate, rectal temperature, and gut motility auscultation. In practice, both behavior observations and physiological measures are commonly obtained concurrently at or in the stall with the patient. Interestingly, authors of a recent review of equine pain assessment [2] proposed a scoring system using only behavior measures based on all studies included in that review. They indicated that physiological measures are less reliable than behavior, as these might be affected by common factors other than pain (e.g., environmental temperature, certain medical conditions, medications including anesthesia, or psychological stress unrelated to pain). They stressed the need that behavior observations be conducted by a familiar trusted person at a distance so as not to disturb the horse. Our observations suggest that for most horses, the familiarity of the individual or the distance within sight or sound does not eliminate the disruption of ongoing discomfort behavior. While caretakers might represent a negative distraction (threat), they might also serve as a positive distraction. For example, it is not unusual to associate people with feeding. In addition, in many equine hospitals, pain evaluations are often done in the morning and evening when other barn activities, representing both positive and negative distractions, typically occur.

Finally, with this in mind, we suggest that equine pain evaluations be conducted remotely via video monitoring, when the hospital environment is otherwise quiet and the horse is undisturbed. Further, we propose that an ideal method would be to obtain a video sample, longer than one might observe in-person, that can be immediately viewed in fast forward to better appreciate frequency and repetitiveness of discomfort behaviors. Video viewing technicians typically learn very quickly how to recognize discomfort behavior when viewing in fast forward. In our lab, new technicians typically achieve acceptable levels of inter-observer reliabilities (r^2^ > 0.80) within a few hours of training. When viewing shorter samples or when viewing in real-time, one might see a limb movement once or twice and not easily recognize it as abnormally repetitive. Similarly, behavior such as restlessness, including frequent interruptions of ongoing goal-directed behavior and fidgeting become more conspicuous in fast forward. A one-hour video sample can be scanned at 10-20X real time, within 5–10 min. The time commitment would be similar to that of a typical stall visit, yet provide greater information.

Another approach to pain recognition and scoring in horses and other animal species is facial grimace evaluation [7,8]. We did not specifically evaluate facial expression in this study, mostly due to inconsistent views of the faces of these patients, but also due to generally poor resolution of the VHS video. Nonetheless, in instances where the horse’s face was in view, we often observed the expression change with the approach of the caretaker and throughout the visit. Studies validating and comparing grimace and composite pain scoring systems typically work from video-recorded samples, while in practice scoring typically involves in-person stall-side evaluation or in-stall caretaker interaction with the patient. In our view, validation should be performed using the method that is used in practice. Remotely observed or recorded high definition still or video facial images might be a more valid method for clinical application of facial grimace scales.

## 5. Conclusions

These findings confirm our clinical impression that caretaker visits interrupt discomfort behavior in horses. Remote assessment will more likely reflect ongoing behavioral signs of discomfor.

## Appendix A

**Table A1:** Discomfort Behaviors Observed Among 20 Orthopedic Surgical Equine Patients.

**Behavioral Indicators of Discomfort**		
***Posture and weight bearing***		
**1. Non-physiologic locomotion**	Lameness, including altered stride, impact, and weight bearing; may include altered limb placement, head and neck movements that suggest off-loading, and/or limited range of motion of a limb	
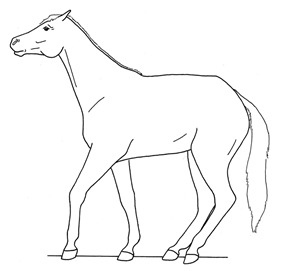
**2. Shifting weight**	Frequent shifting of the primary weight bearing limb or limbs	
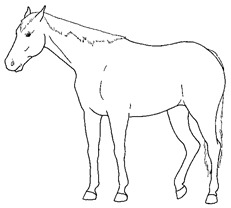
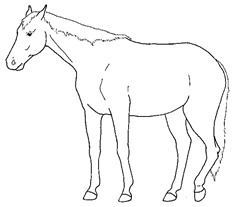
**3. Pointing**	Consistent extension of one forelimb, reducing weight bearing on that limb	
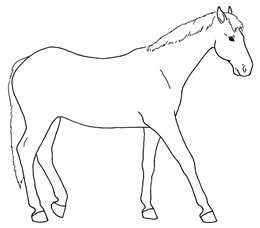
**4. Prolonged resting of limb**	Flexing a fore or hind limb, resting the toe of the hoof on the substrate for a prolonged period	
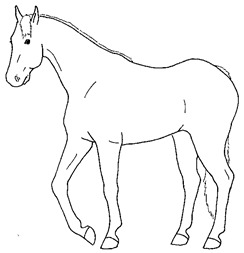
**5. Base narrow or wide**	Standing or moving with fore and/or hind limbs placed either more medially or laterally than is normal for the horse’s conformation	
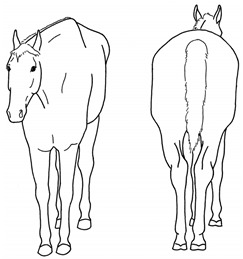
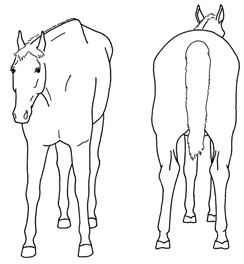
**6. Leaning against objects**	Supporting weight and/or stabilizing balance against a wall or fence, usually during standing rest	
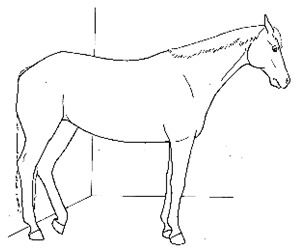
**7. Atypical recumbency**	Prolonged or frequent interrupted recumbent rests and/or increased recumbent resting time budget	
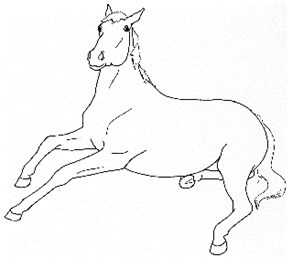
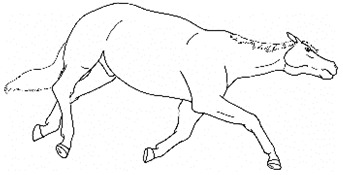
**8. Difficulty rising**	Failing to rise gracefully, requiring increased effort and/or attempts to rise	
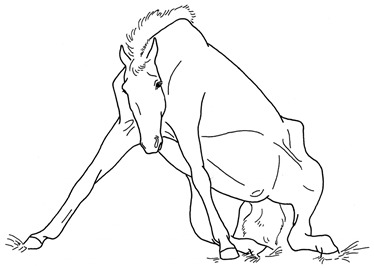
***Limb and body movements***		
**9. Stepping in place**	Repeated flexing of a limb, briefly relieving weight bearing on that limb	
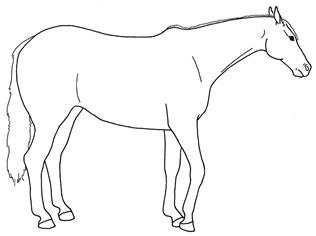
**10. Lifting/holding limb up**	Flexing a limb, lifting the hoof off of the substrate (usually 20cm or more) and then placing it back down	
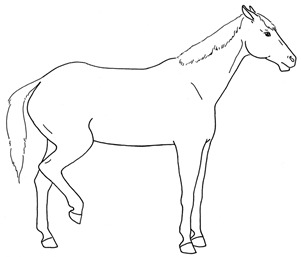
**11. Pawing**	Reaching a forelimb cranially and dragging the hoof along or above the substrate while sweeping the limb caudally, often in rhythmic series	
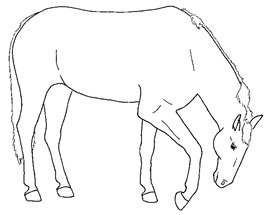
**12. Kicking out or back**	Lifting and extending one or both hind limbs caudally, either straight back or arching laterally	
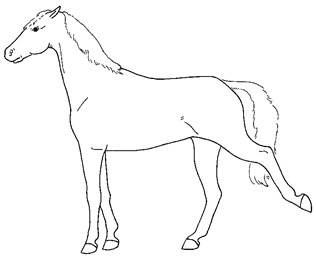
**13. Romping/Bucking**	Alternate rearing and kicking out, often repeatedly in rapid succession	
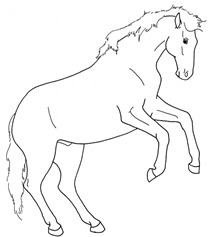
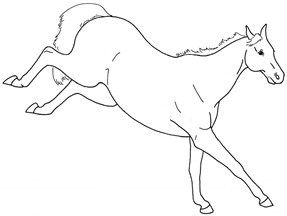
**14. Rolling**	Lying down to sternal recumbency and then rotating from sternal to dorsal recumbency, sometimes from one side to the other	
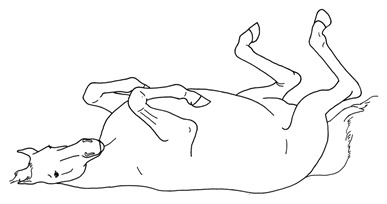
**15. Flinching**	Sudden reflexive contraction of muscles	
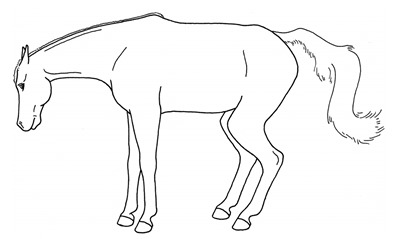
**16. Stretching**	Extending or straightening part of the body to its full extent, can take multiple forms	
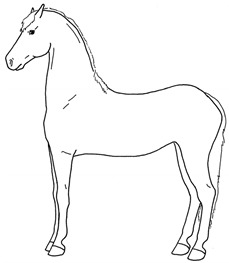
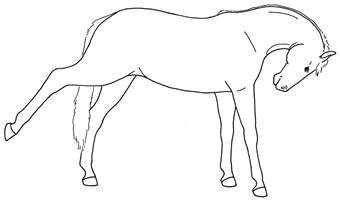
***Head, neck, mouth, and lip movements***		
**17. Rotational shaking head or whole body**	Rapid, rhythmic rotation of the whole body or just the head and neck along the long axis	
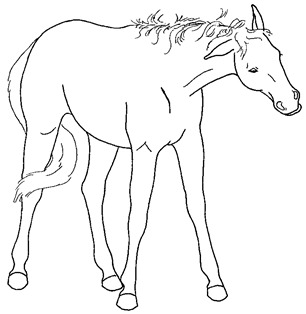
**18. Frustration head tossing**	Quick rotational toss of the head, similar to a head threat	
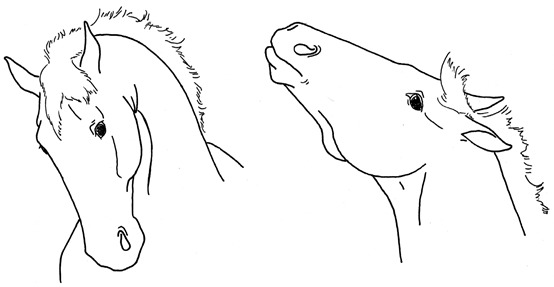
**19. Head bobbing**	Repetitive rhythmic nodding of the head and neck	
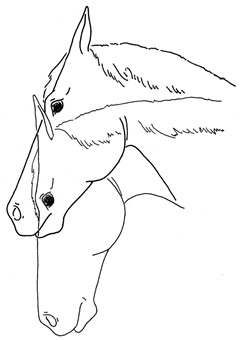
**20. Abbreviated weaving**	Rhythmic side-to-side swaying of the head and neck	
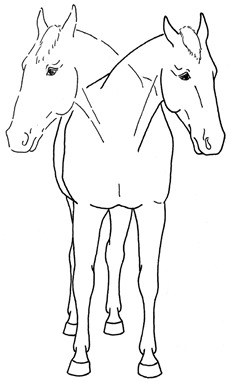
**21. Sympathetic surge resolution signs: extending tongue, licking, chewing, itching**	Cluster of autonomic responses following an acute sympathetic surge, including salivation (leading to chewing movements, swallowing, tongue extensions) and or/autogrooming (typically rubbing face against forelimb)	
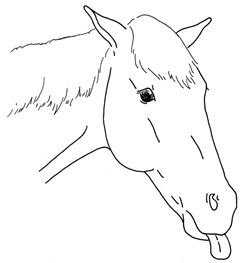
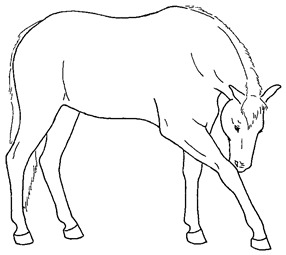
**22. Frequent yawning bouts**	Frequent bouts of yawning, often with a greater number of yawns per bout than is characteristic (>3–5)	
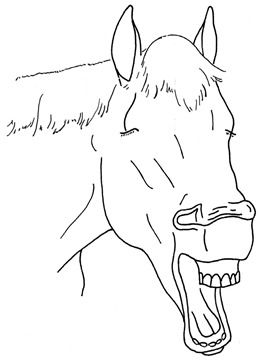
***Attention to an area***		
**23. Looking**	Repeated glancing or gazing at a particular area of the body	
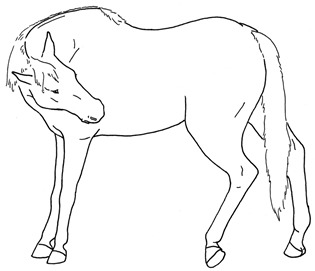
**24. Swatting**	Swinging the head and neck to bat at a particular area of the body	
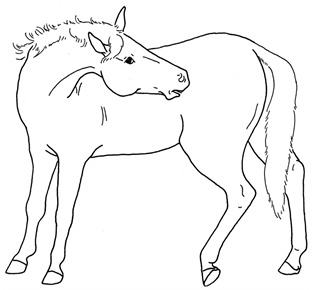
**25. Autogrooming**	Nibbling, nuzzling, biting at and area of the body, or rubbing one part of the body against another or against an object	
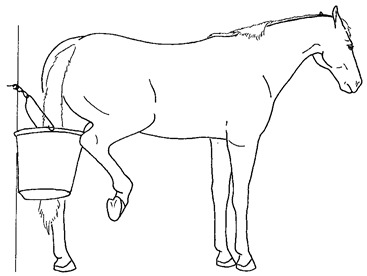
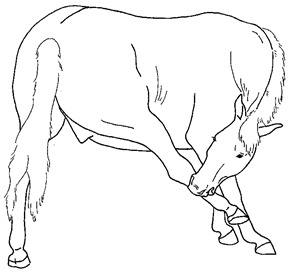
***Ear and tail movements***		
**26. Moving/focusing ear caudally**	Rotating ears to focus caudally, or laying ears back against neck	
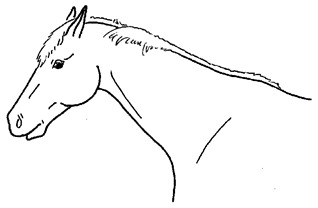
**27.Swishing/flicking tail**	Moving tail suddenly from side to side	
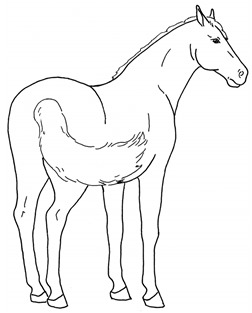
***Overall demeanor***		
**28. Changing activities frequently**	Uncharacteristically changing major activity (foraging, standing rest, standing alert) more frequently than expected, as often as from minute to minute with other accompanying signs of discomfort	
**29. Conservative movement**	Less than typical movement about the stall, with apparent hesitation to walk	
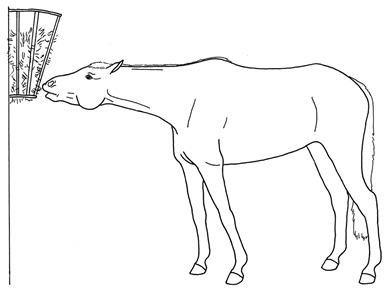
**30. Circling/Pacing**	Walking in circle usually along a perimeter	
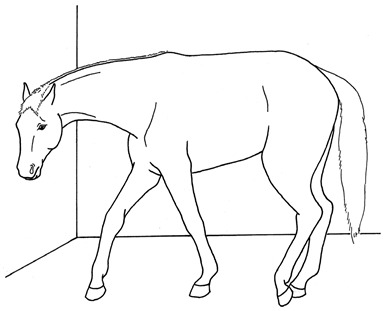
**31. Abandoning recumbency or elimination attempts**	Repeated posturing as if intending to lie down, urinate, or defecate that appears interrupted by discomfort	
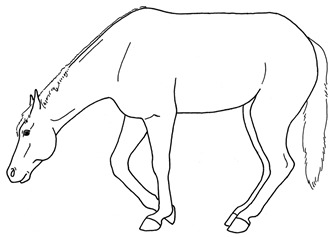
**32. Nibbling aimlessly**	Reduced interest in hay or feed, but continued nominal foraging gestures, often directed at non-food objects	
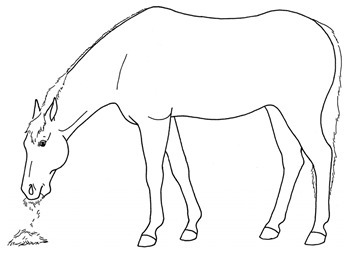
**33. Fidgeting**	Biting, mouthing, or rubbing against objects (e.g., stall walls, feed/water containers)	
